# Editorial for the Special Issue on Fundamentals and Applications of Micro/Nanorobotics

**DOI:** 10.3390/mi15111303

**Published:** 2024-10-27

**Authors:** Chunyun Wei, Zhuoran Zhang, Xian Wang, Haojian Lu, Jiangfan Yu

**Affiliations:** 1 School of Science and Engineering, The Chinese University of Hong Kong, Shenzhen 518172, China; weichunyun@tju.edu.cn; 2 School of Chemical Engineering and Technology, Tianjin University, Tianjin 300350, China; 3 Department of Mechanical and Materials Engineering, Queen’s University, Kingston, ON K7L 3N6, Canada; 4 State Key Laboratory of Industrial Control and Technology, Zhejiang University, Hangzhou 310027, China

## 1. Introduction to This Special Issue

In recent years, microrobots have drawn extensive attention due to their promising potential in biomedical applications [1], especially for on-demand targeted drug delivery [2], hyperthermia [3], remote sensing [4], and biopsy [5].Miniaturized microrobots and responsive materials have enabled complex and precise operations in the living body, thus starting a revolution in healthcare [1].

There is a significant amount of previous research on the fundamentals and applications of microrobots, and remarkable advancements have been made. Different power sources have been investigated to actuate microrobots, such as magnetic field [6,7], optical field [8,9], electric field [10], chemical energy [11], and acoustic field [12]. Meanwhile, soft microrobots [13,14,15], helical microrobots [16,17,18], microrobot swarms [19,20], and biohybrid microrobots have been investigated [21,22,23]. Automatic control algorithms have been applied [24,25,26] for the motion control of microrobots. The high-precision navigation of microrobots has been facilitated by deploying control methods [27,28,29,30]. Challenges also exist in designing microrobots, including new material inventions [31,32], process control [33,34], a fast and precise fabrication protocol [35], and adaptation to physiological environments [36]. Therefore, the field of microrobotics still requires research efforts to develop from fundamentals to applications. This Special Issue concentrates on the fundamentals and applications of microrobots, including innovative designs [37,38], control methods [39,40,41,42], and their potential applications. The progress in and limitations of microneedles relating to delivery approaches, parameters involved, and biocompatibility [43] are also included.

**Figure 1:**
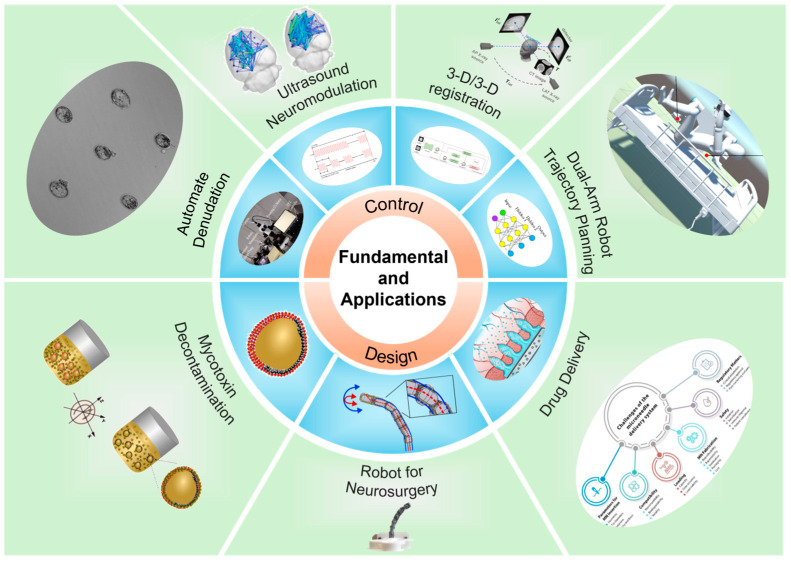
Schematic illustration of the topics in this Special Issue on fundamentals and applications of micro/nanorobotics.

As shown in Figure 1, this Special Issue comprises five research papers, one communication paper, and one review paper, and one article was selected as the Editor’s choice. Each of the articles is briefly summarized in the following section.

## 2. Designs Proposed in This Special Issue

The challenges of microrobots could be tackled by applying a “design-by-apply” approach [44]. The designs proposed in this Special Issue all are from practical biomedical application problems, including two different types of designs, such as microrobots based on rolling joints [37] and biohybrid microrobots [38]. In addition, a review about the progress and challenges of microneedles was included [43].

Rolling joints can improve motion control in cable-driven continuum robots. Dragone et al. [37] presented a minimally invasive manipulator characterized by hyper-redundant kinematics and embedded sensing modules. An optical fiber was embedded in the robot body to measure the attenuation of the light transmitted, and the angles at the distal extremity of the robot can thus be estimated. This manipulator enabled the real-time measurement, curvature control, and tip bending of continuum robots. Moreover, the authors evaluated the sensing and control performance of the robot during the bending of the tip.

For microrobots fabricated by integrating cells with functional materials, Lu et al. [38] designed Janus yeast cell microrobots for mycotoxin decontamination. In their research, Janus yeast cell microrobots (JYC-robots) were fabricated by integrating Fe_3_O_4_ nanoparticles (MNPs) for magnetically driving and the in situ growth of zeolitic imidazolate framework-67 (ZIF-67) for mycotoxin removal. The result indicated that the wrapped MNPs accelerate the toxin-removal efficacy of JYC-robots. Furthermore, the authors then made comparisons of the motion performance levels and toxin-removal efficacies between JYC-robots and yeast cells fully coated with Fe_3_O_4_ NPs and ZIF-67 (FC-yeasts). The experimental results indicated that although FC-yeasts showed a faster motion than JYC-robots, the latter had a higher efficiency of zearalenone (ZEN) removal. The results verified the significance of the yeast cell wall in toxin removal. This asymmetrical modification method simultaneously realized magnetic actuation and toxin-removal efficacy.

Avcil and Çelik [43] systematically reviewed the literature on microneedles in drug delivery. A microneedle (MN)-mediated delivery system is denoted as the non-invasive delivery of medications through the skin surface via transdermal drug delivery (TDD) methods, drawing much attention from researchers. This technique makes pain-free drug delivery possible because it has been verified that an MN can penetrate the viable epidermis of the skin and bypass the stratum corneum. In the past few decades, researchers have proposed many microneedle-based delivery approaches, such as poke and patch via solid MNs, coat and poke via coated MNs, poke and flow via hollow MNs, poke and dissolve via dissolving MNs, and poke and release via hydrogel-forming MNs. However, there are still some challenges in translating this technology into real applications, and the possible solutions identified could determine the future of the field and its commercial applications. Avcil and Çelik’s review summarized the existing microneedle-based delivery approaches and provided an outlook based on the parameters of an MN system, compatibility, loading, fabrication, safety, and regulatory matters.

## 3. Control Methods Discussed in This Special Issue

This Special Issue concentrates on two cases of the automatic control of microrobots, including the focused ultrasound-mediated control [40] and the automated denudation of oocytes [39]. In addition, deep reinforcement learning (DRL) was applied to the control methods, such as 2-D/3-D rigid registration [42] for highly accurate control, and trajectory planning based on DRL [41] for complex environments control.

Zhang et al. [40] compared two different modes of low-intensity focused ultrasound in the attenuation of epilepsy. The main idea of this research was to employ two modes of low-intensity focused ultrasound, pulsed wave (pw), and continuous wave (cw), with a 0.5 MHZ center frequency for the kainic acid (KA)-induced epileptic rats. In their work, a 32-channel EEG electrode was used to obtain EEG signals, and the power spectral density (PSD) was calculated to analyze the epileptic situation. The authors found that ultrasound neuromodulation effectively regulates epileptic brain connections, and the effects of these two modes of ultrasound on epileptic brain network were similar. The presented ultrasound-mediated region-specific functional neuromodulation promises to become an effective method for studying brain function and neurological diseases. The effect of performing ultrasound on network connections is also a potentially new research direction for the mechanism of ultrasound neuromodulation.

Zhai et al. [39] introduced a robotic system for automated oocyte denudation. In their system, the visual feedback regarding the positions of the micropipette tip, oocytes, and the surrounding cumulus cells was used to form an image-based visual servo control system. Moreover, a contact detection algorithm, filling algorithm, and automated calibration algorithm were deployed to identify the tip, fill the holes caused by the varied image intensity of oocyte surface parts, and calibrate the XY stage, respectively. The experiments indicate that the error of the automated oocyte denudation system was lower than 5 μm, whereas the error of the manual method was higher than that. Furthermore, this system could achieve a higher yield rate and denudation efficiency than the manual method, for which the rate was 97.0 ± 2.8% and the efficiency was 95.0 ± 0.8%. The results demonstrated the potential in clinical intracytoplasmic sperm injection.

Tang et al. [41] proposed a method to plan an optimized trajectory for navigating two machine arms autonomously in complex environments based on a deep reinforcement learning (DRL). A trial of the motion of the human body in bed was investigated. A neural network with a proximal policy optimization (PPO) algorithm was trained, and a continuous reward function was applied to facilitate robot maneuvering. The authors also proposed reward and punishment functions inspired by the artificial potential field. The simulation results indicated that the PPO algorithm reduces the number of steps required for the training to converge by about 4 million fewer than is required by the DDPG algorithm. Moreover, the function enabled the robots to obtain a higher reward than other reward and punishment functions. This proposed method thus has the advantage of interactions between humans and robots.

For intra-operative target pose estimation, An et al. [42] proposed a novel orthogonal-view 2-D/3-D rigid registration framework. The main idea of their research was the combination of deep-learning-based dense reconstruction with the 3-D/3-D registration. The dense reconstruction transferred the 2-D/3-D registration into the registration of two images with the same dimension (3-D), which avoided the ill-posed nature and dimensional reduction. In addition, the parallelization strategy and GPU were used to accelerate the 3-D/3-D registration. The authors focused on radiofrequency ablation (RFA) in trigeminal neuralgia to verify the performance of this framework and used the CT-DRR dataset to evaluate the proposed method. The experimental results showed that the method achieved a mean target registration error of 1.65 ± 1.41 mm, a 20% gross failure rate, and a 1.8 s running time, which were better than those of the state-of-the-art approach POINT^2^+opt.

## 4. Applications Discussed in This Special Issue

The articles in this Special Issue show potential applications for micro/nanorobotics in biomedicine, especially approaching hard-to-reach regions in the human body.

For the design of micro/nanorobotics, magnetic-propelled Janus yeast cell robots proposed by Lu et al. [38] are expected to achieve environment remediation. In addition, this coupling of MNPs and the metal–organic framework method could easily be extended to different types of cells to achieve other applications [45,46]. An embedded-sensing continuum robot fabricated via 3-D-printing is proposed by Dragone et al.37, and is expected to contribute to the automation of neurosurgical tasks and minimally invasive surgery [47]. The review entitled “Microneedles in Drug Delivery: Progress and Challenges” [43] contributes by transferring microneedles from lab benches to clinics.

For the control of micro/nanorobotics, the article “Different Modes of Low-Frequency Focused Ultrasound-Mediated Attenuation of Epilepsy Based on the Topological Theory” [40] provided insights for non-invasive treatments. An automated oocyte denudation system has been reported by Zhai et al. [39] and is expected to feature in the applications of automated human samples processing. Dual-arm robot trajectory planning based on DRL [41] shows the potential of capturing and estimating human–robotic arm interactions. Moreover, this method will further contribute to the magnetic actuation systems for micro/nanorobots accessing hard-to-reach sites [48]. The robust orthogonal-view 2-D/3-D rigid registration [42] can be applied to tasks that demand high accuracy.

## 5. Conclusions

In conclusion, this Special Issue highlights the cutting-edge achievements in micro/nanorobotics from fundamentals to applications. It also reveals the major challenges in this area: The first challenge is to optimize the design of micro/nanorobots. Innovative designs for micro/nanorobots with high motion dexterity and multi-functions are demanded to achieve complex tasks in vivo. Secondly, a precise closed-loop control is still critical for accurate task execution. In this Special Issue, different algorithms were proposed to improve control accuracy and rapidity. In complex in vivo environments, various algorithms should be customized to guarantee the completion of the given tasks.

We would like to take this opportunity to thank all authors who have contributed to this Special Issue. In addition, we appreciate each reviewer for their professional and efficient review reports for the submitted manuscripts. We finally thank the Editorial Office of *Micromachines* for their support in this Special Issue.

## Data Availability

Some data are available from the corresponding authors upon request.

## References

[1] Li J., Esteban-Fernández de Ávila B., Gao W., Zhang L., Wang J. (2017). Micro/nanorobots for biomedicine: Delivery, surgery, sensing, and detoxification. Sci. Robot..

[2] Luo M., Feng Y., Wang T., Guan J. (2018). Micro-/nanorobots at work in active drug delivery. Adv. Funct. Mater..

[3] Choi J., Hwang J., Kim J.Y., Choi H. (2021). Recent progress in magnetically actuated microrobots for targeted delivery of therapeutic agents. Adv. Healthc. Mater..

[4] Wang Q., Yang S., Zhang L. (2024). Untethered micro/nanorobots for remote sensing: Toward intelligent platform. Nano-Micro Lett..

[5] Vikram Singh A., Sitti M. (2016). Targeted drug delivery and imaging using mobile milli/microrobots: A promising future towards theranostic pharmaceutical design. Curr. Pharm. Des..

[6] Chen H., Wang Y., Liu Y., Zou Q., Yu J. (2022). Sensing of fluidic features using colloidal microswarms. ACS Nano.

[7] Yu J., Jin D., Chan K.-F., Wang Q., Yuan K., Zhang L. (2019). Active generation and magnetic actuation of microrobotic swarms in bio-fluids. Nat. Commun..

[8] Gao Y., Guo Y., Yang Y., Tang Y., Wang B., Yan Q., Chen X., Cai J., Fang L., Xiong Z. (2024). Magnetically Manipulated Optoelectronic Hybrid Microrobots for Optically Targeted Non-Genetic Neuromodulation. Adv. Mater..

[9] Iványi G.T., Nemes B., Gróf I., Fekete T., Kubacková J., Tomori Z., Bánó G., Vizsnyiczai G., Kelemen L. (2024). Optically Actuated soft Microrobot Family for Single-cell Manipulation. Adv. Mater..

[10] Diwakar N.M., Kunti G., Miloh T., Yossifon G., Velev O.D., Science I. (2022). AC electrohydrodynamic propulsion and rotation of active particles of engineered shape and asymmetry. Curr. Opin. Colloid Interface Sci..

[11] Zheng C., Lin J., Song X., Gan Q., Lin X. (2022). TiO_2_-nanoparticle-shelled light-driven microcleaner for fast and highly efficient degradation of organic pollutants. ACS Appl. Nano Mater..

[12] Deng Y., Paskert A., Zhang Z., Wittkowski R., Ahmed D. (2023). An acoustically controlled helical microrobot. Sci. Adv..

[13] Hu W., Lum G.Z., Mastrangeli M., Sitti M. (2018). Small-scale soft-bodied robot with multimodal locomotion. Nature.

[14] Liu X., Wang L., Xiang Y., Liao F., Li N., Li J., Wang J., Wu Q., Zhou C., Yang Y. (2024). Magnetic soft microfiberbots for robotic embolization. Sci. Robot..

[15] Dong X., Xiao B., Vu H., Lin H., Sitti M. (2024). Millimeter-scale soft capsules for sampling liquids in fluid-filled confined spaces. Sci. Adv..

[16] Peyer K.E., Tottori S., Qiu F., Zhang L., Nelson B.J. (2013). Magnetic helical micromachines. Chem. –A Eur. J..

[17] Nam J., Lee W., Kim J., Jang G. (2017). Magnetic helical robot for targeted drug-delivery in tubular environments. IEEE/ASME Trans. Mechatron..

[18] Ghadami S., Shum H. (2024). Designing a magnetic micro-robot for transporting stiff filamentous microcargo. Phys. Fluids.

[19] Yu J., Wang B., Du X., Wang Q., Zhang L. (2018). Ultra-extensible ribbon-like magnetic microswarm. Nat. Commun..

[20] Wang Q., Wang Q., Ning Z., Chan K.F., Jiang J., Wang Y., Su L., Jiang S., Wang B., Ip B.Y.M. (2024). Tracking and navigation of a microswarm under laser speckle contrast imaging for targeted delivery. Sci. Robot..

[21] Felfoul O., Mohammadi M., Taherkhani S., De Lanauze D., Zhong Xu Y., Loghin D., Essa S., Jancik S., Houle D., Lafleur M. (2016). Magneto-aerotactic bacteria deliver drug-containing nanoliposomes to tumour hypoxic regions. Nat. Nanotechnol..

[22] Park S.J., Park S.-H., Cho S., Kim D.-M., Lee Y., Ko S.Y., Hong Y., Choy H.E., Min J.J., Park J.O. (2013). New paradigm for tumor theranostic methodology using bacteria-based microrobot. Sci. Rep..

[23] Akolpoglu M.B., Alapan Y., Dogan N.O., Baltaci S.F., Yasa O., Aybar Tural G., Sitti M. (2022). Magnetically steerable bacterial microrobots moving in 3D biological matrices for stimuli-responsive cargo delivery. Sci. Adv..

[24] Tottori S., Zhang L., Qiu F., Krawczyk K.K., Franco-Obregón A., Nelson B.J. (2012). Magnetic helical micromachines: Fabrication, controlled swimming, and cargo transport. Adv. Mater..

[25] Kummer M.P., Abbott J.J., Kratochvil B.E., Borer R., Sengul A., Nelson B.J. (2010). OctoMag: An electromagnetic system for 5-DOF wireless micromanipulation. IEEE Trans. Robot..

[26] Gauri H.M., Patel R., Lombardo N.S., Bevan M.A., Bharti B. (2024). Field-Directed Motion, Cargo Capture, and Closed-Loop Controlled Navigation of Microellipsoids. Small.

[27] Liu Y., Chen H., Zou Q., Du X., Wang Y., Yu J. (2023). Automatic navigation of microswarms for dynamic obstacle avoidance. IEEE Trans. Robot..

[28] Yang L., Yu J., Zhang L. (2019). Statistics-based automated control for a swarm of paramagnetic nanoparticles in 2-D space. IEEE Trans. Robot..

[29] Yu J., Xu T., Lu Z., Vong C.I., Zhang L. (2017). On-demand disassembly of paramagnetic nanoparticle chains for microrobotic cargo delivery. IEEE Trans. Robot..

[30] Kim H., Kim M.J. (2015). Electric field control of bacteria-powered microrobots using a static obstacle avoidance algorithm. IEEE Trans. Robot..

[31] Chen C., Ding S., Wang J. (2024). Materials consideration for the design, fabrication and operation of microscale robots. Nat. Rev. Mater..

[32] Schmidt C.K., Medina-Sánchez M., Edmondson R.J., Schmidt O.G. (2020). Engineering microrobots for targeted cancer therapies from a medical perspective. Nat. Commun..

[33] Wang M., Wu T., Liu R., Zhang Z., Liu J. (2023). Selective and Independent Control of Microrobots in a Magnetic Field: A Review. Engineering.

[34] Wang Y., Chen H., Xie L., Liu J., Zhang L., Yu J. (2024). Swarm Autonomy: From Agent Functionalization to Machine Intelligence. Adv. Mater..

[35] Dabbagh S.R., Sarabi M.R., Birtek M.T., Seyfi S., Sitti M., Tasoglu S. (2022). 3D-printed microrobots from design to translation. Nat. Commun..

[36] Zhao S., Sun D., Zhang J., Lu H., Wang Y., Xiong R., Grattan K.T.V. (2022). Actuation and biomedical development of micro-/nanorobots—A review. Mater. Today Nano.

[37] Dragone D., Donadio F.F., Mirabelli C., Cosentino C., Amato F., Zaffino P., Spadea M.F., La Torre D., Merola A. (2023). Design and Experimental Validation of a 3D-Printed Embedded-Sensing Continuum Robot for Neurosurgery. Micromachines.

[38] Lu D., Tang S., Li Y., Cong Z., Zhang X., Wu S. (2021). Magnetic-propelled Janus yeast cell robots functionalized with metal-organic frameworks for mycotoxin decontamination. Micromachines.

[39] Zhai R., Shan G., Dai C., Hao M., Zhu J., Ru C., Sun Y. (2022). Automated Denudation of Oocytes. Micromachines.

[40] Zhang M., Li B., Liu Y., Tang R., Lang Y., Huang Q., He J. (2021). Different modes of low-frequency focused ultrasound-mediated attenuation of epilepsy based on the topological theory. Micromachines.

[41] Tang W., Cheng C., Ai H., Chen L. (2022). Dual-arm robot trajectory planning based on deep reinforcement learning under complex environment. Micromachines.

[42] An Z., Ma H., Liu L., Wang Y., Lu H., Zhou C., Xiong R., Hu J. (2021). Robust orthogonal-view 2-D/3-D rigid registration for minimally invasive surgery. Micromachines.

[43] Avcil M., Çelik A. (2021). Microneedles in drug delivery: Progress and challenges. Micromachines.

[44] Lee J.G., Raj R.R., Day N.B., Shields IV C.W. (2023). Microrobots for biomedicine: Unsolved challenges and opportunities for translation. ACS Nano.

[45] Sadeghi A., Ebrahimi M., Shahryari S., Assadpour E., Jafari S.M. (2024). Potential applications of encapsulated yeasts especially within alginate and chitosan as smart bioreactors and intelligent micro-machines. Carbohydr. Polym. Technol. Appl..

[46] Li J., Dekanovsky L., Khezri B., Wu B., Zhou H., Sofer Z. (2022). Biohybrid micro-and nanorobots for intelligent drug delivery. Cyborg. Bionic Syst..

[47] Shi C., Luo X., Qi P., Li T., Song S., Najdovski Z., Fukuda T., Ren H. (2016). Shape sensing techniques for continuum robots in minimally invasive surgery: A survey. IEEE Trans. Biomed. Eng..

[48] Yang Z., Yang H., Cao Y., Cui Y., Zhang L. (2023). Magnetically actuated continuum medical robots: A review. Adv. Intell. Syst..

